# Supplementary material for: Computational neural dynamics of goal-directed visual attention in macaques
Source: Sci Adv. 2026 May 20;12(21):eaed9004. doi: 10.1126/sciadv.aed9004 (PMC13189128; doi:10.1126/sciadv.aed9004)
Supplement: Supplementary file 1 — Figs. S1 to S5 [file sciadv.aed9004_sm.pdf]

Science Advances
AAAS

# Supplementary Materials for

## Computational neural dynamics of goal-directed visual attention in macaques

Jie Zhang *et al.*

Corresponding author: Jie Zhang, zjie@wustl.edu; Shuo Wang, shuowang@wustl.edu

*Sci. Adv.* **12**, eaed9004 (2026)
DOI: 10.1126/sciadv.aed9004

**This PDF file includes:**

Figs. S1 to S5

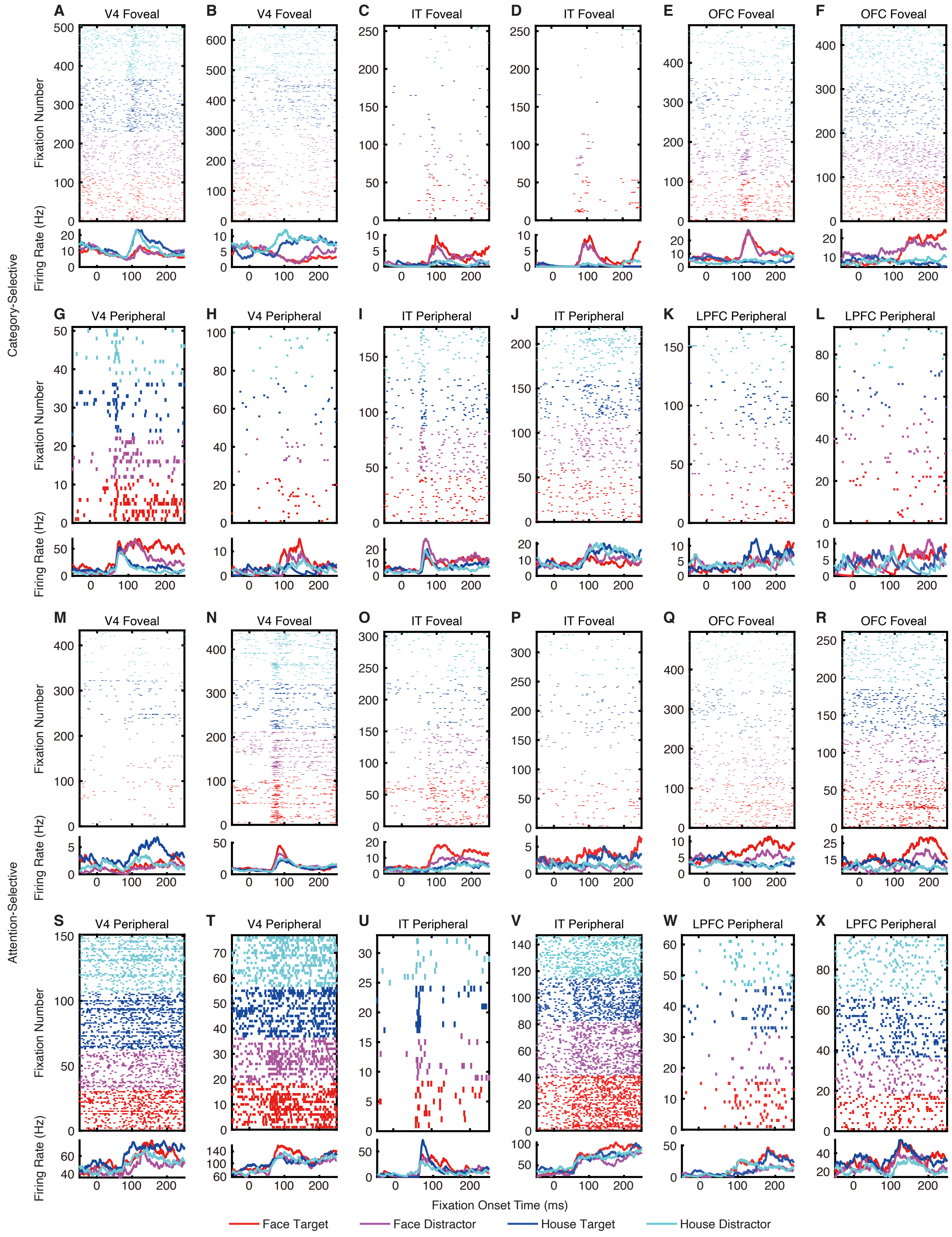

Category-Selective
Attention-Selective
A V4 Foveal
B V4 Foveal
C IT Foveal
D IT Foveal
E OFC Foveal
F OFC Foveal
G V4 Peripheral
H V4 Peripheral
I IT Peripheral
J IT Peripheral
K LPFC Peripheral
L LPFC Peripheral
M V4 Foveal
N V4 Foveal
O IT Foveal
P IT Foveal
Q OFC Foveal
R OFC Foveal
S V4 Peripheral
T V4 Peripheral
U IT Peripheral
V IT Peripheral
W LPFC Peripheral
X LPFC Peripheral
Fixation Number
Firing Rate (Hz)
Fixation Onset Time (ms)
Face Target
Face Distractor
House Target
House Distractor

**fig. S1. Additional results for category-selective and attention-selective units. (A–F)** Category-selective foveal units. **(G–L)** Category-selective peripheral units. **(M–R)** Attention-selective foveal units. **(S–X)** Attention-selective peripheral units. **(A, B, G, H, M, N, S, T)** V4 units. **(C, D, I, J, O, P, U, V)** IT units. **(E, F, Q, R)** OFC units. **(K, L, W, X)** LPFC units. Legend conventions as in **Fig. 1**.

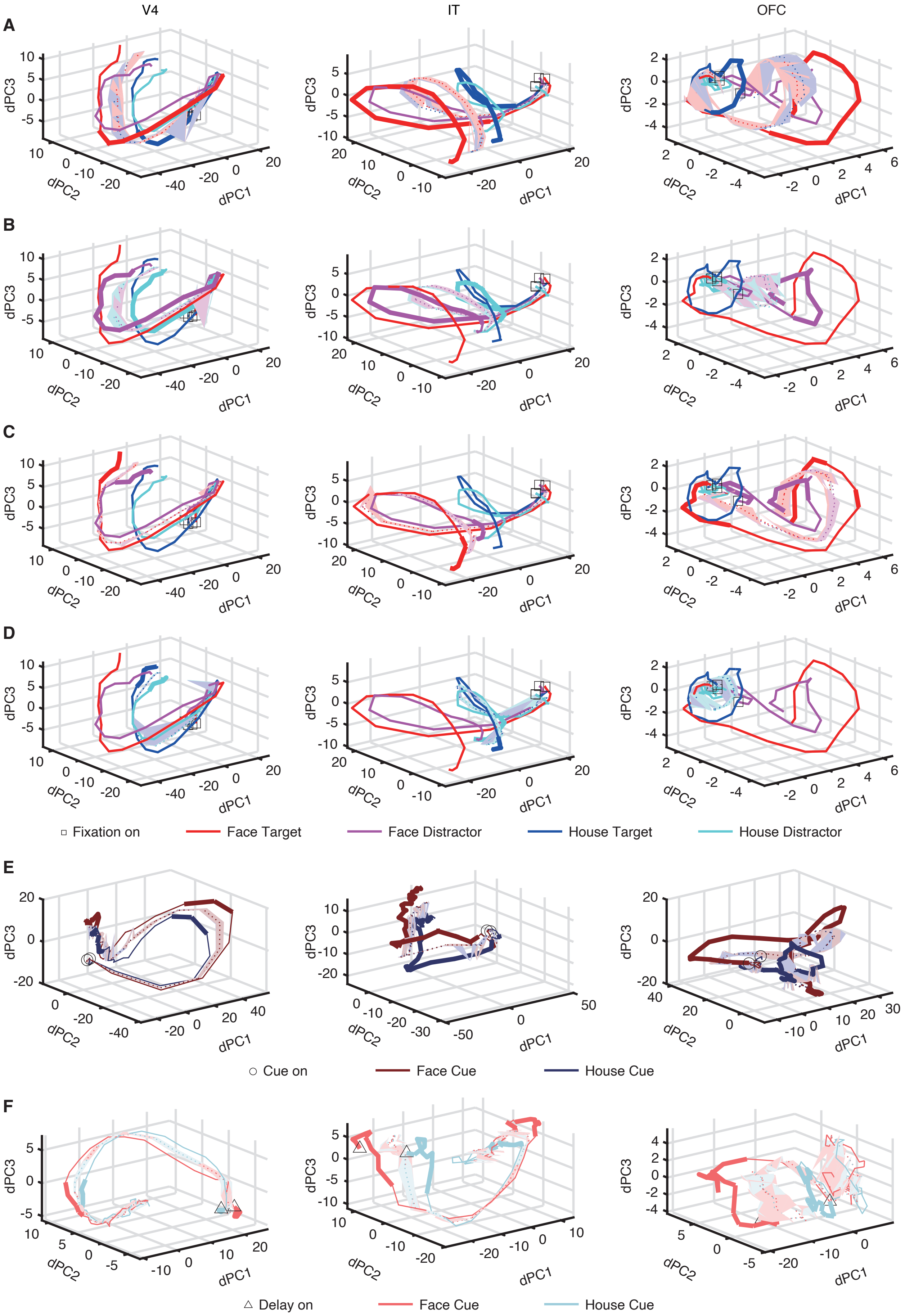

V4
IT
OFC
A
B
C
D
dPC1
dPC2
dPC3
Fixation on
Face Target
Face Distractor
House Target
House Distractor
E
Cue on
Face Cue
House Cue
F
Delay on
Face Cue
House Cue

**fig. S2. Control results for neural population dynamics. (A–D)** Statistical comparison using permutation tests for each brain area in the main (category-matching) task. **(A)** Comparison of face targets versus house targets. **(B)** Comparison of face versus house distractors. **(C)** Comparison of face targets versus face distractors. **(D)** Comparison of house targets versus house distractors. **(E, F)** Replication of results in the identity-matching task. In this task, there was only one search target, and monkeys were required to fixate on the identical target matching the cue. Legend conventions as in **Fig. 2**.

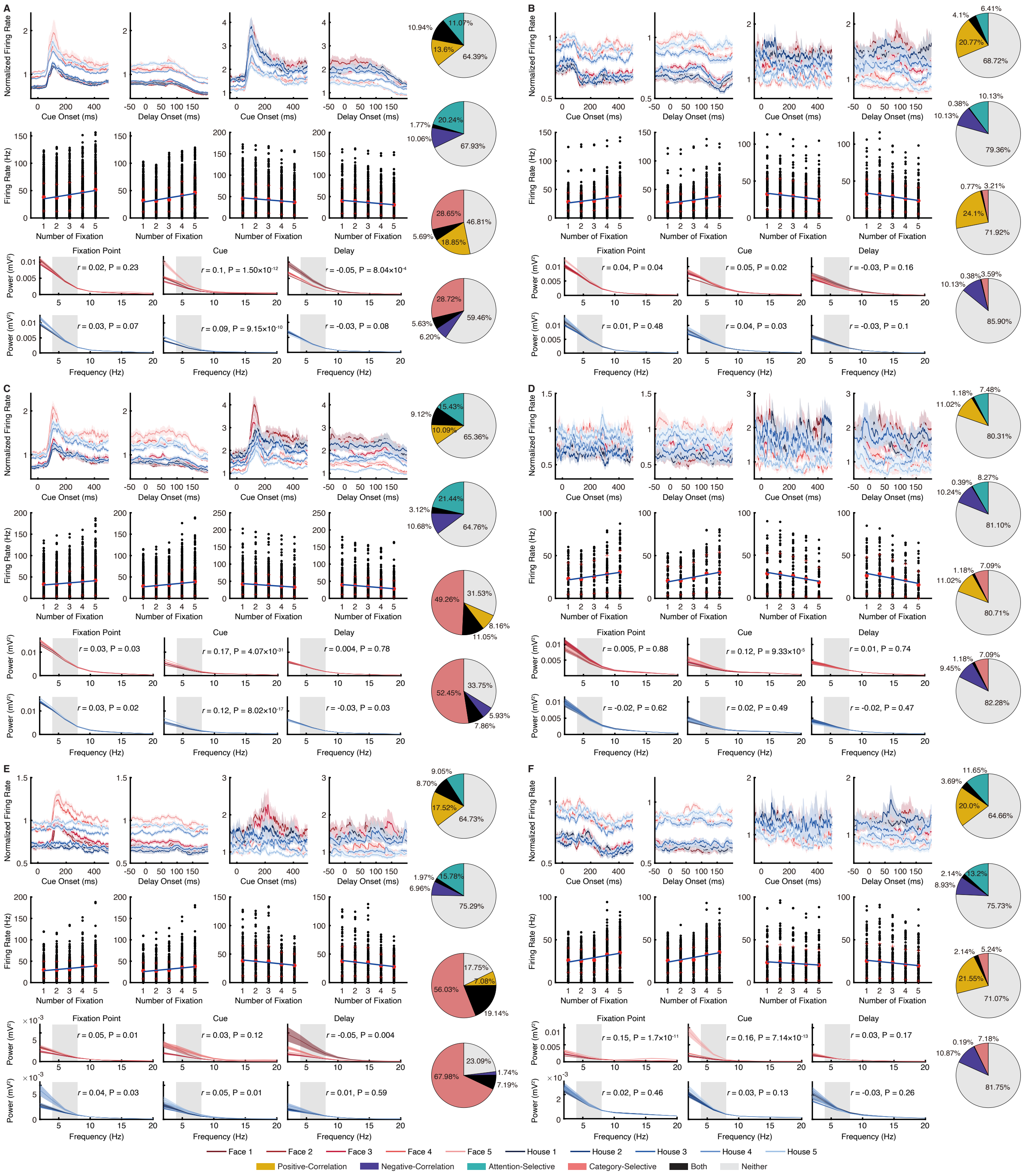


**fig. S3. Neural activity during cue presentation and maintenance, analyzed separately for foveal and peripheral units.** **(A)** V4 foveal units. **(B)** V4 peripheral units. **(C)** IT foveal units. **(D)** IT peripheral units. **(E)** OFC foveal units. **(F)** LPFC peripheral units. Legend conventions as in **Fig. 3**.

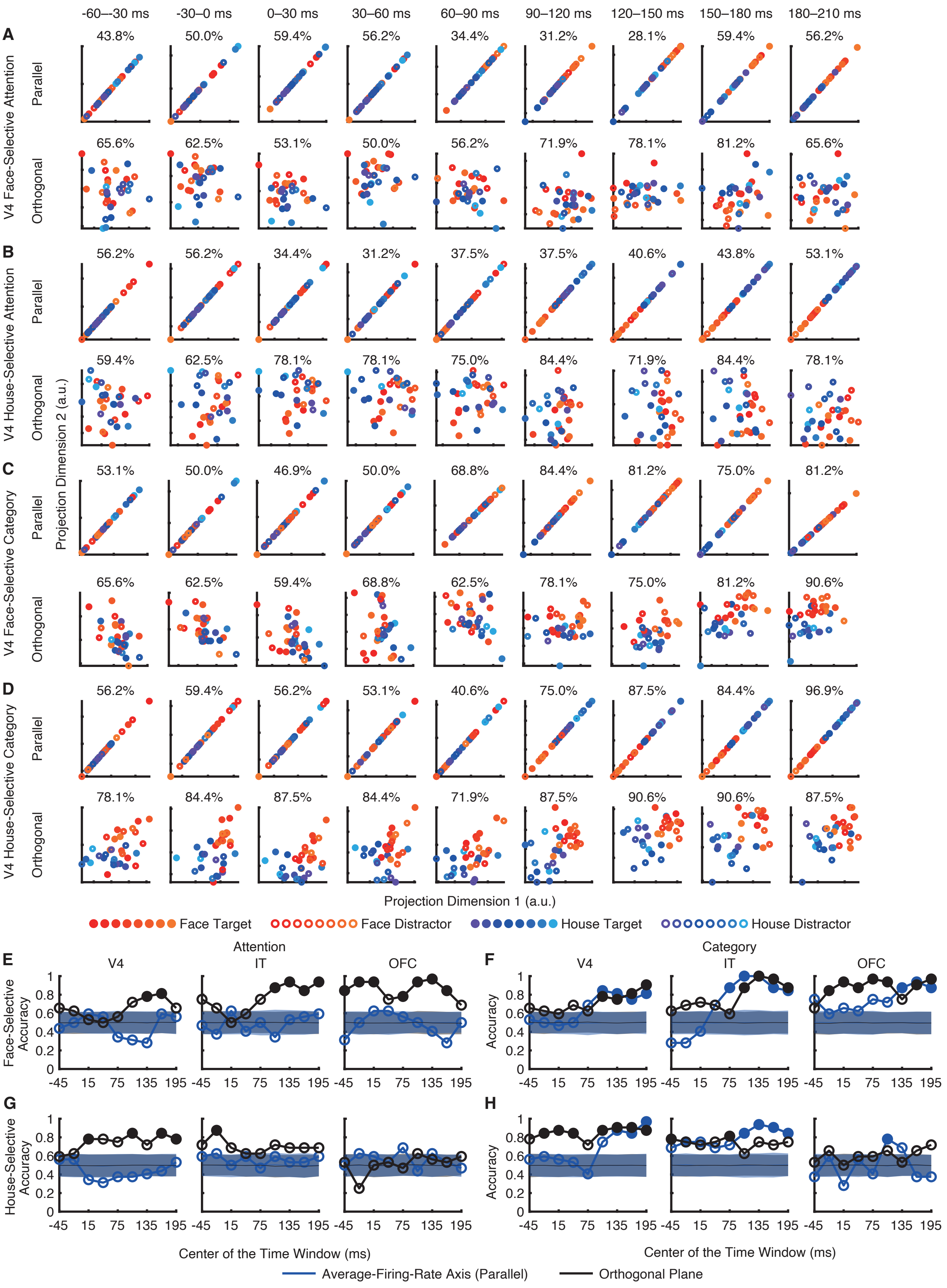

-60–-30 ms
-30–0 ms
0–30 ms
30–60 ms
60–90 ms
90–120 ms
120–150 ms
150–180 ms
180–210 ms
A
V4 Face-Selective Attention
Parallel
43.8% 50.0% 59.4% 56.2% 34.4% 31.2% 28.1% 59.4% 56.2%
Orthogonal
65.6% 62.5% 53.1% 50.0% 56.2% 71.9% 78.1% 81.2% 65.6%
B
V4 House-Selective Attention
Parallel
56.2% 56.2% 34.4% 31.2% 37.5% 37.5% 40.6% 43.8% 53.1%
Orthogonal
59.4% 62.5% 78.1% 78.1% 75.0% 84.4% 71.9% 84.4% 78.1%
C
V4 Face-Selective Category
Parallel
53.1% 50.0% 46.9% 50.0% 68.8% 84.4% 81.2% 75.0% 81.2%
Orthogonal
65.6% 62.5% 59.4% 68.8% 62.5% 78.1% 75.0% 81.2% 90.6%
D
V4 House-Selective Category
Parallel
56.2% 59.4% 56.2% 53.1% 40.6% 75.0% 87.5% 84.4% 96.9%
Orthogonal
78.1% 84.4% 87.5% 84.4% 71.9% 87.5% 90.6% 90.6% 87.5%
Projection Dimension 2 (a.u.)
Projection Dimension 1 (a.u.)
Face Target
Face Distractor
House Target
House Distractor
Attention
Category
E
F
V4
IT
OFC
Face-Selective Accuracy
Accuracy
G
H
House-Selective Accuracy
1
0.8
0.6
0.4
0.2
0
-45 15 75 135 195
Center of the Time Window (ms)
Average-Firing-Rate Axis (Parallel)
Orthogonal Plane

**fig. S4. Neural processing across stages of visual search in the orthogonal subspace for face-selective and house-selective units.** Projection of the population response of V4 units onto the attention subspace **(A, B)** and category subspace **(C, D)**. **(E, G)** Classification accuracy for attention. **(F, H)** Classification accuracy for category. **(A, C, E, F)** Face-selective units. **(B, D, G, H)** House-selective units. Legend conventions as in **Fig. 4**.

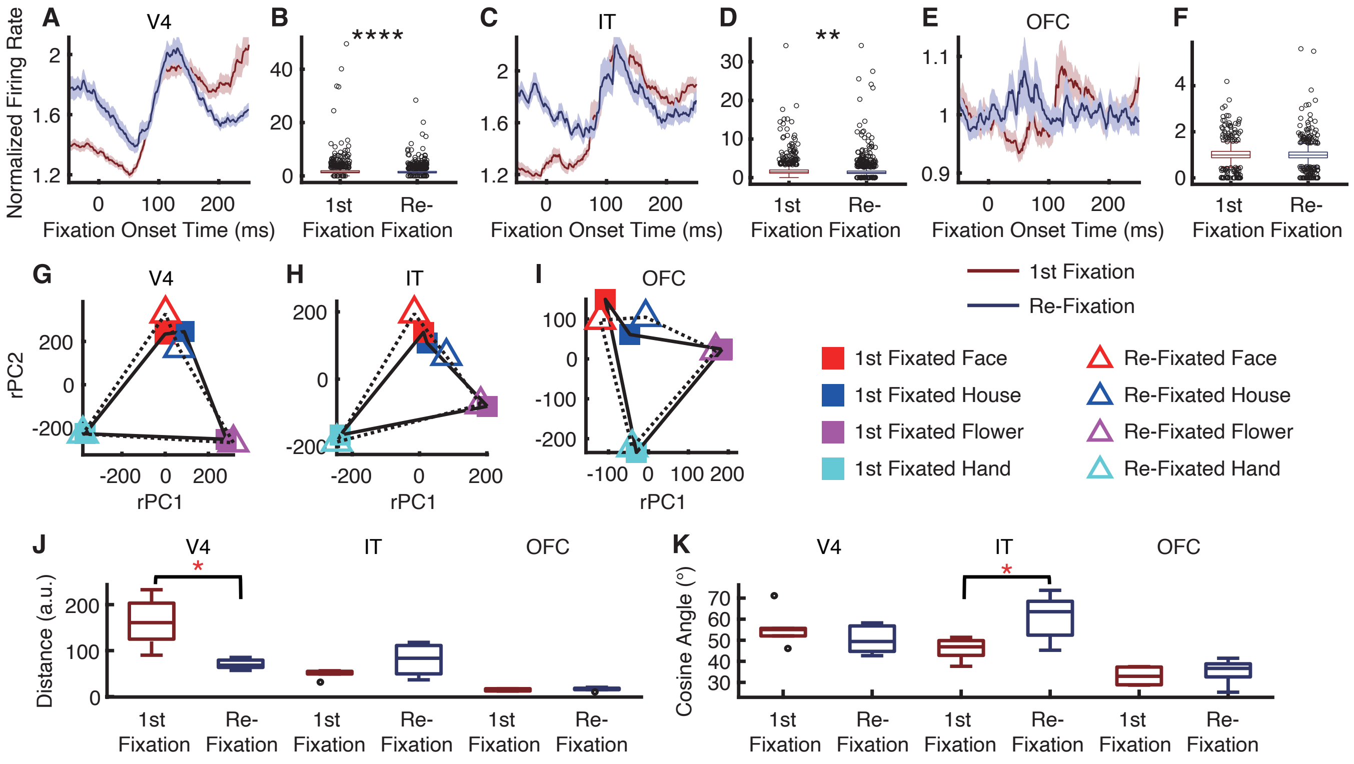


**fig. S5. Comparison of first-fixated versus re-fixated search items using only distractor stimuli. (A, B, G)** V4 units. **(C, D, H)** IT units. **(E, F, I)** OFC units. **(A, C, E)** Mean normalized firing rate. **(B, D, F)** Mean normalized firing rate in a time window 150–250 ms after fixation onset. **(G–I)** Projection of the population response onto the neural state space. **(J)** Representational distance for the population of units. **(K)** Angle between neuronal vectors. Legend conventions as in **Fig. 6**.
